# The genome sequence of the Grey Chi, *Antitype chi* (Linnaeus, 1761)

**DOI:** 10.12688/wellcomeopenres.19288.1

**Published:** 2023-04-06

**Authors:** David Lees, Natural History Museum Genome Acquisition Lab, Darwin Tree of Life Barcoding collective, Wellcome Sanger Institute Tree of Life programme, Wellcome Sanger Institute Scientific Operations: DNA Pipelines collective, Tree of Life Core Informatics collective, Darwin Tree of Life Consortium

**Affiliations:** 1 Natural History Museum, London, England, UK

**Keywords:** Antitype chi, Grey Chi, genome sequence, chromosomal, Lepidoptera

## Abstract

We present a genome assembly from an individual male *Antitype chi* (the Grey Chi; Arthropoda; Insecta; Lepidoptera; Noctuidae). The genome sequence is 632.2 megabases in span. Most of the assembly is scaffolded into 31 chromosomal pseudomolecules, including the assembled Z sex chromosome. The mitochondrial genome has also been assembled and is 15.3 kilobases in length.

## Species taxonomy

Eukaryota; Metazoa; Ecdysozoa; Arthropoda; Hexapoda; Insecta; Pterygota; Neoptera; Endopterygota; Lepidoptera; Glossata; Ditrysia; Noctuoidea; Noctuidae; Xyleninae; *Antitype*; *Antitype chi* (Linnaeus, 1761) (NCBI:txid988062).

## Background

The Grey Chi, *Antitype chi*, is a medium sized noctuid moth, whitish-grey background reticulated with darker markings including a distinctive black mark likened to an anvil or Greek letter χ in the forewing. These patterns camouflage it on walls and rocks, and semi-melanic forms occur, supposedly adaptive to dark substrates. The moth is monovoltine and flies in August and September in the UK, overwintering as an egg ( Waring *et al.*, 2017).

The Grey Chi is found in moorlands and grassy hillsides in the uplands. The larva appears to be quite polyphagous, feeding on leaves of various shrubs such as *Crataegus* and *Ribes*, and, in captivity, has been fed on a wide variety of low growing plants such as *Rumex* ( Waring *et al.*, 2017).

*Antitype chi* is generally common and widespread in the western Palaearctic only, from southern Scandinavia to the shores of the Mediterranean, with scattered records into Russia ( GBIF Secretariat, 2022). In the UK ( NBN Atlas Partnership, 2021), it is rather local, and rare or vagrant in south-eastern England, relatively common towards the north ( Waring *et al*., 2017) with records concentrated in northern and western areas. However, populations in the UK have shown an overall decrease of 57% between 1970–2016 ( Fox *et al.*, 2021), affecting both abundance and distribution ( Randle *et al.*, 2019).

The genus *Antitype* (Hübner, 1821) is currently placed in the noctuid tribe Xylenini, with five congeners. It is quite close on BOLD (within about 5.5% pairwise divergence in COI-5P) to genera such as *Leucochlaena* Hampson, 1906 and *Polymixis* (Hübner, 1820) but it has apparently not yet been included in molecular phylogenetic works, so the genome of *A. chi* will be very useful for evolutionary studies.

The genome sequence should not only be useful in phylogeny but in studies of potentially cryptic species. There are essentially two DNA barcode clusters on BOLD (10 March 2023): the BINs BOLD:AAE7040 (most exemplars including the UK ones), and BOLD:ADF3730 (two records only from Germany and Norway which differ by just one base; these are about 1.12% pairwise divergent from BOLD:AAE7040). A closely related cluster (BOLD:AAM0440) from the Southern Europe, about 2.5% pairwise divergent from BOLD:AAE7040, is otherwise identified as *A. suda* (Geyer, 1832) or *A. jonis* (Lederer, 1865) .

For discussion of the controversy about alleged industrial melanism in the Grey Chi see ( Fryer, 2013) and the website of historian Alan Brooke.

The genome of *Antitype chi* was sequenced as part of the Darwin Tree of Life Project, a collaborative effort to sequence all named eukaryotic species in the Atlantic Archipelago of Britain and Ireland. Here we present a chromosomally complete genome sequence for *Antitype chi*, based on one male specimen from Beinn Eighe National Nature Reserve, Scotland.

### Genome sequence report

**Figure 1.**
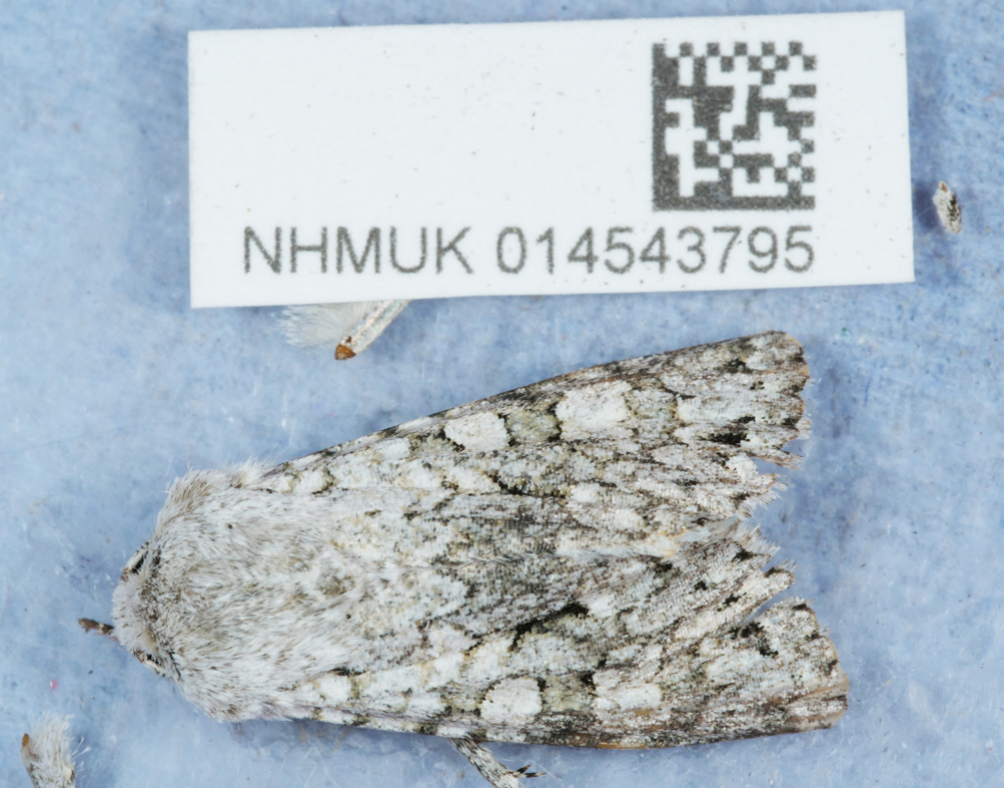
Photograph of the *Antitype chi* (ilAntChix2) specimen used for genome sequencing.

The genome was sequenced from one male *Antitype chi* ( Figure 1) collected from Beinn Eighe National Nature Reserve, Scotland, UK (latitude 57.63, longitude –5.35). A total of 25-fold coverage in Pacific Biosciences single-molecule HiFi long reads was generated. Primary assembly contigs were scaffolded with chromosome conformation Hi-C data. Manual assembly curation corrected 13 missing or mis-joins and removed three haplotypic duplications, reducing the scaffold number by 7.32%, and increasing the scaffold N50 by 1.11%.

**Figure 2.**
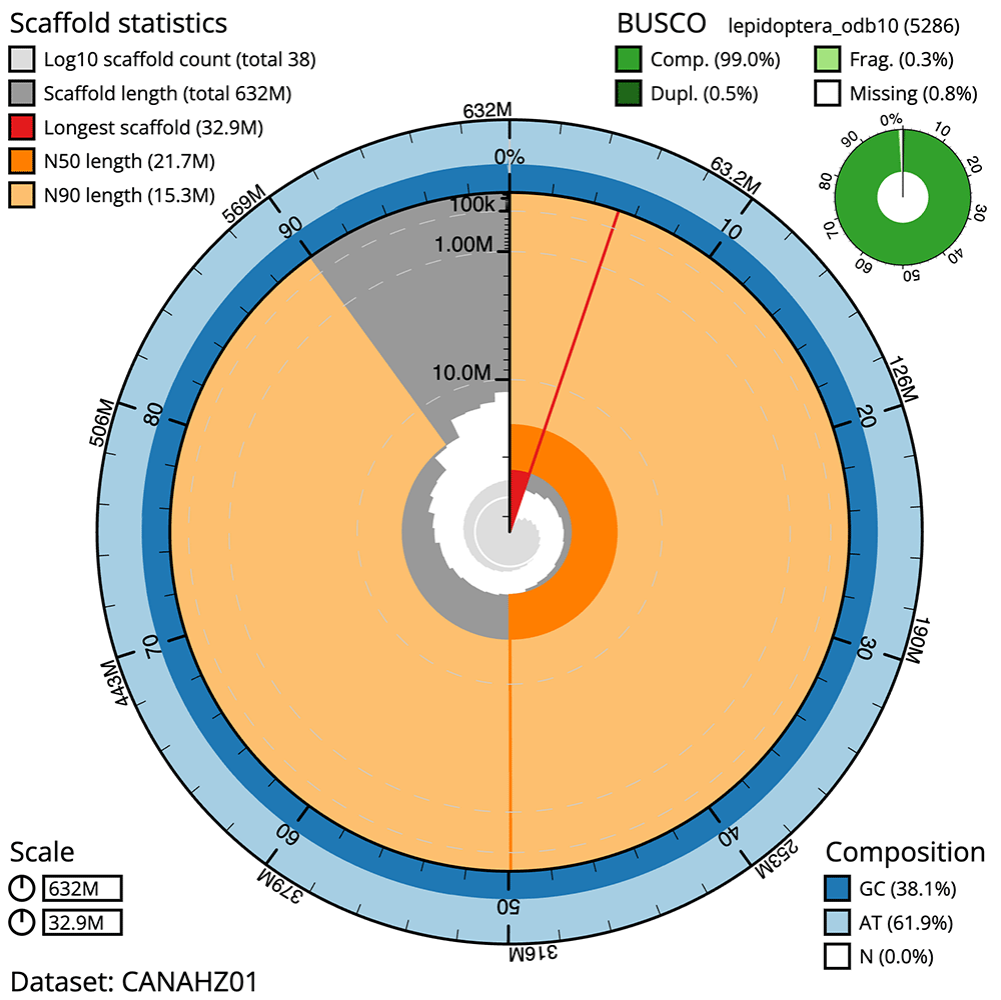
Genome assembly of *Antitype chi*, ilAntChix2.1: metrics. The BlobToolKit Snailplot shows N50 metrics and BUSCO gene completeness. The main plot is divided into 1000 size-ordered bins around the circumference with each bin representing 0.1% of the 632,218,267 bp assembly. The distribution of scaffold lengths is shown in dark grey with the plot radius scaled to the longest scaffold present in the assembly (32,898,968 bp, shown in red). Orange and pale-orange arcs show the N50 and N90 sequence lengths (21,733,341 and 15,314,028 bp), respectively. The pale grey spiral shows the cumulative scaffold count on a log scale with white scale lines showing successive orders of magnitude. The blue and pale-blue area around the outside of the plot shows the distribution of GC, AT and N percentages in the same bins as the inner plot. A summary of complete, fragmented, duplicated and missing BUSCO genes in the lepidoptera_odb10 set is shown in the top right. An interactive version of this figure is available at https://blobtoolkit.genomehubs.org/view/ilAntChix2.1/dataset/CANAHZ01/snail.

The final assembly has a total length of 632.2 Mb in 38 sequence scaffolds with a scaffold N50 of 21.7 Mb ( Table 1). Most (99.98%) of the assembly sequence was assigned to 31 chromosomal-level scaffolds, representing 30 autosomes, and the Z sex chromosome. Chromosome-scale scaffolds confirmed by the Hi-C data are named in order of size ( Figure 2– Figure 5; Table 2). While not fully phased, the assembly deposited is of one haplotype. Contigs corresponding to the second haplotype have also been deposited. The mitochondrial genome was also assembled and can be found as a contig within the multifasta file of the genome submission.

**Table 1.** Genome data for *Antitype chi*, ilAntChix2.1.

Project accession data		
Assembly identifier	ilAntChix2.1	
Species	*Antitype chi*	
Specimen	ilAntChix2	
NCBI taxonomy ID	988062	
BioProject	PRJEB55575	
BioSample ID	SAMEA110028560	
Isolate information	ilAntChix2; head and thorax (genome sequencing and Hi-C scaffolding), abdomen (RNA sequencing)	
Assembly metrics *		*Benchmark*
Consensus quality (QV)	66.3	*≥ 50*
*k*-mer completeness	100%	*≥ 95%*
BUSCO **	C:99.0%[S:98.4%,D:0.5%], F:0.3%,M:0.8%,n:5,286	*C ≥ 95%*
Percentage of assembly mapped to chromosomes	99.98%	*≥ 95%*
Sex chromosomes	Z chromosome	*localised* *homologous* *pairs*
Organelles	Mitochondrial genome assembled, 15.3 kb, OX375838.1	*complete single* *alleles*
Raw data accessions		
PacificBiosciences SEQUEL II	ERR10115641	
Hi-C Illumina	ERR10123714	
PolyA RNA-Seq Illumina	ERR10890705	
Genome assembly		
Assembly accession	GCA_947359405.1	
*Accession of alternate* *haplotype*	GCA_947359415.1	
Span (Mb)	632.2	
Number of contigs	109	
Contig N50 length (Mb)	10.4	
Number of scaffolds	38	
Scaffold N50 length (Mb)	21.7	
Longest scaffold (Mb)	32.9	

* Assembly metric benchmarks are adapted from column VGP-2020 of “Table 1: Proposed standards and metrics for defining genome assembly quality” from ( Rhie *et al.*, 2021). ** BUSCO scores based on the lepidoptera_odb10 BUSCO set using v5.3.2. C = complete [S = single copy, D = duplicated], F = fragmented, M = missing, n = number of orthologues in comparison. A full set of BUSCO scores is available at https://blobtoolkit.genomehubs.org/view/ilAntChix2.1/dataset/CANAHZ01/busco.

**Figure 3.**
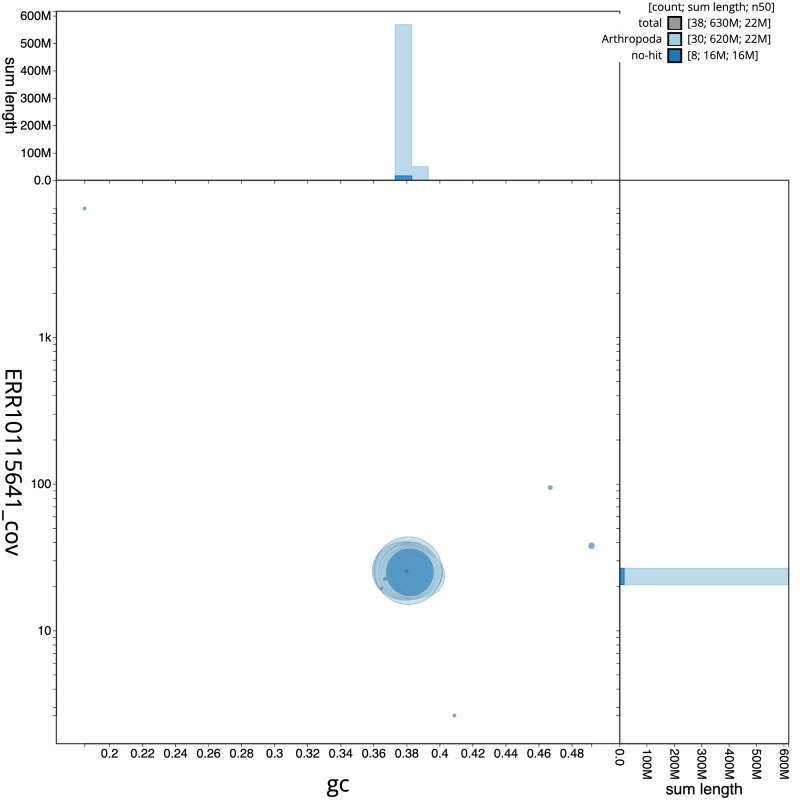
Genome assembly of *Antitype chi*, ilAntChix2.1: GC coverage. BlobToolKit GC-coverage plot. Scaffolds are coloured by phylum. Circles are sized in proportion to scaffold length. Histograms show the distribution of scaffold length sum along each axis. An interactive version of this figure is available at https://blobtoolkit.genomehubs.org/view/ilAntChix2.1/dataset/CANAHZ01/blob.

**Figure 4.**
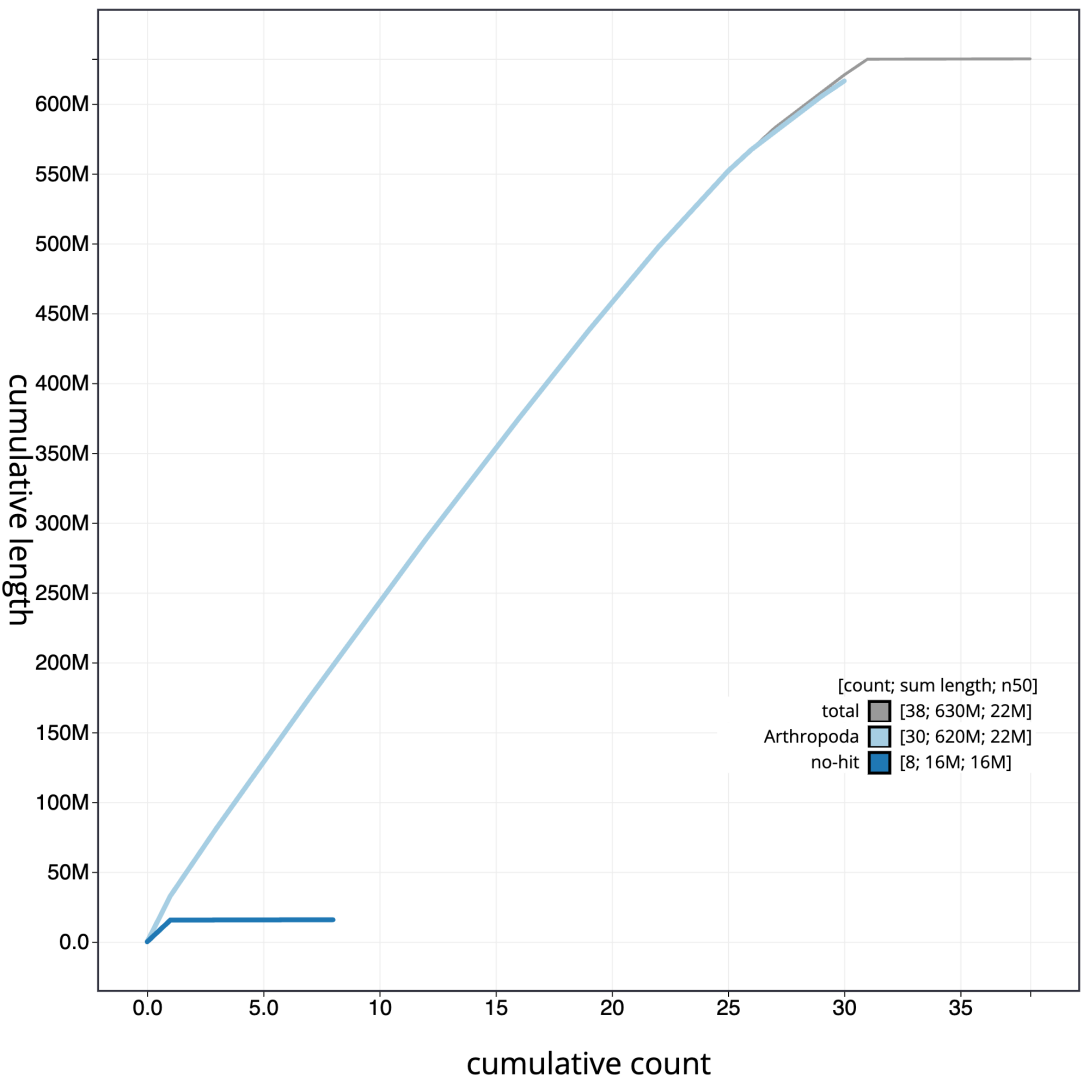
Genome assembly of *Antitype chi*, ilAntChix2.1. BlobToolKit cumulative sequence plot. The grey line shows cumulative length for all scaffolds. Coloured lines show cumulative lengths of scaffolds assigned to each phylum using the buscogenes taxrule. An interactive version of this figure is available at https://blobtoolkit.genomehubs.org/view/ilAntChix2.1/dataset/CANAHZ01/cumulative.

**Figure 5.**
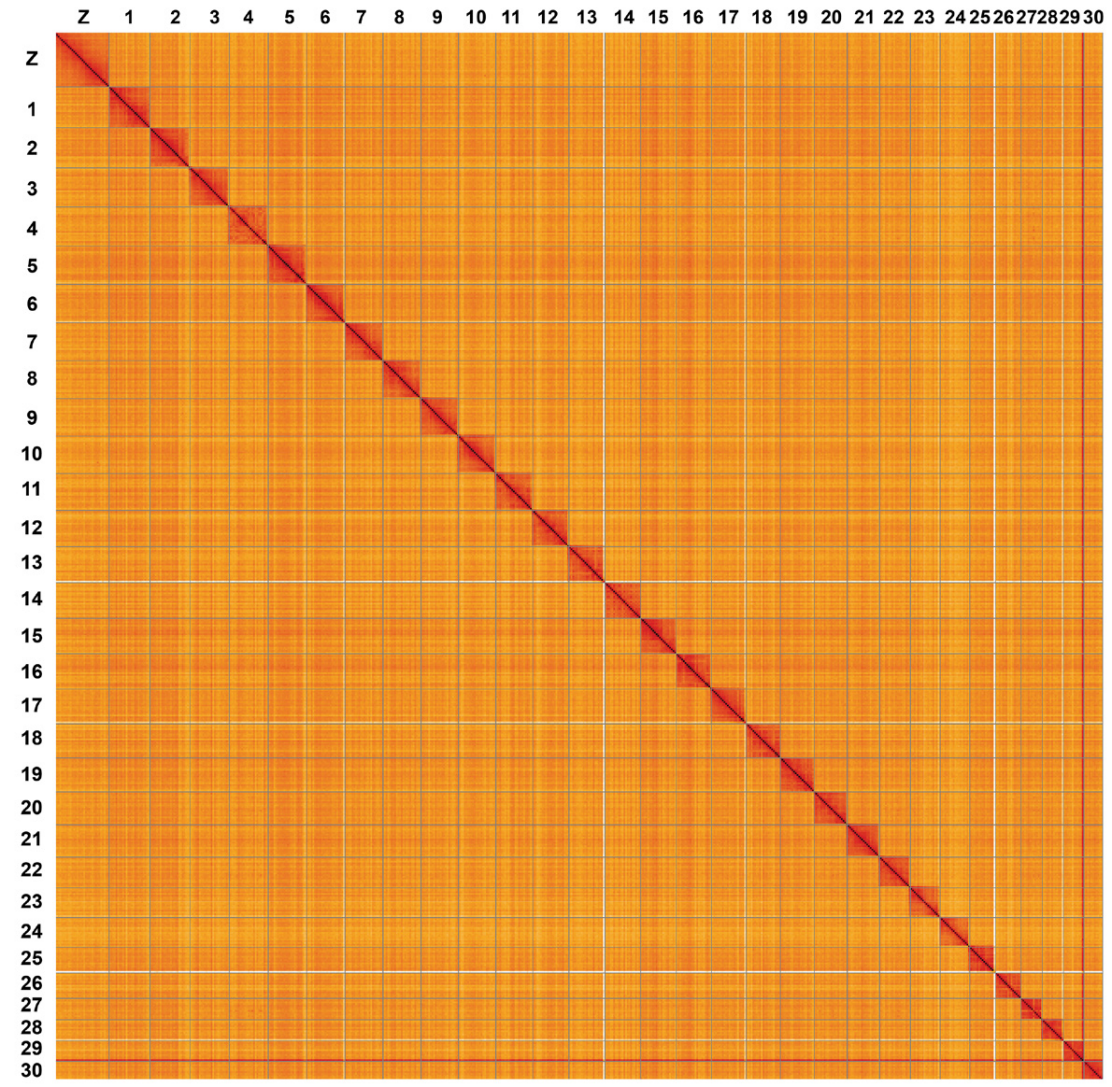
Genome assembly of *Antitype chi*, ilAntChix2.1. Hi-C contact map of the ilAntChix2.1 assembly, visualised using HiGlass. Chromosomes are shown in order of size from left to right and top to bottom. An interactive version of this figure may be viewed at https://genome-note-higlass.tol.sanger.ac.uk/l/?d=ZP2Z6P9aSIK1F9Zxa2AEKA.

**Table 2.** Chromosomal pseudomolecules in the genome assembly of *Antitype chi*, ilAntChix2.

INSDC accession	Chromosome	Size (Mb)	GC%
OX375808.1	1	24.65	38
OX375809.1	2	24.21	38.2
OX375810.1	3	23.58	37.8
OX375811.1	4	23.51	38.1
OX375812.1	5	23.2	38.2
OX375813.1	6	23.1	38.2
OX375814.1	7	22.92	38.1
OX375815.1	8	22.88	37.7
OX375816.1	9	22.63	37.9
OX375817.1	10	22.53	37.9
OX375818.1	11	22.28	37.8
OX375819.1	12	21.85	37.8
OX375820.1	13	21.73	37.6
OX375821.1	14	21.5	38
OX375822.1	15	21.37	38
OX375823.1	16	21.32	38
OX375824.1	17	21	38.2
OX375825.1	18	20.55	38.3
OX375826.1	19	20.48	38.1
OX375827.1	20	19.81	37.9
OX375828.1	21	19.51	38.3
OX375829.1	22	18.55	38.3
OX375830.1	23	17.99	38.1
OX375831.1	24	17.82	38.2
OX375832.1	25	15.57	38.2
OX375833.1	26	15.31	37.9
OX375834.1	27	12.87	39
OX375835.1	28	12.76	38.4
OX375836.1	29	12.33	38.9
OX375837.1	30	11.26	38.5
OX375807.1	Z	32.9	38.1
OX375838.1	MT	0.02	18.8
-	unplaced	0.22	46.1

The estimated Quality Value (QV) of the final reference assembly is 66.3 with *k*-mer based completeness of 100%, and the assembly has a BUSCO v5.3.2 completeness of 99.0% (single = 98.4%, duplicated = 0.5%), using the lepidoptera_odb10 reference set ( *n* = 5,286).

Metadata for specimens, spectral estimates, sequencing runs, contaminants and pre-curation assembly statistics can be found here.

## Methods

### Sample acquisition and nucleic acid extraction

A male *Antitype chi* (specimen number NHMUK014543795, ToLID ilAntChix2) was collected from Beinn Eighe National Nature Reserve, Scotland, UK (latitude 57.63, longitude –5.35) on 10 September 2021. The specimen was collected by David Lees (Natural History Museum) using a light trap. The specimen was identified by the collector and dry-frozen at –80°C.

DNA was extracted at the Tree of Life laboratory, Wellcome Sanger Institute (WSI). The ilAntChix2 sample was weighed and dissected on dry ice with tissue set aside for Hi-C sequencing. Head and thorax tissue was disrupted using a Nippi Powermasher fitted with a BioMasher pestle. High molecular weight (HMW) DNA was extracted using the Qiagen MagAttract HMW DNA extraction kit. HMW DNA was sheared into an average fragment size of 12–20 kb in a Megaruptor 3 system with speed setting 30. Sheared DNA was purified by solid-phase reversible immobilisation using AMPure PB beads with a 1.8X ratio of beads to sample to remove the shorter fragments and concentrate the DNA sample. The concentration of the sheared and purified DNA was assessed using a Nanodrop spectrophotometer and Qubit Fluorometer and Qubit dsDNA High Sensitivity Assay kit. Fragment size distribution was evaluated by running the sample on the FemtoPulse system.

RNA was extracted from abdomen tissue of ilAntChix2 in the Tree of Life Laboratory at the WSI using TRIzol, according to the manufacturer’s instructions. RNA was then eluted in 50 μL RNAse-free water and its concentration assessed using a Nanodrop spectrophotometer and Qubit Fluorometer using the Qubit RNA Broad-Range (BR) Assay kit. Analysis of the integrity of the RNA was done using Agilent RNA 6000 Pico Kit and Eukaryotic Total RNA assay.

### Sequencing

Pacific Biosciences HiFi circular consensus DNA sequencing libraries were constructed according to the manufacturers’ instructions. Poly(A) RNA-Seq libraries were constructed using the NEB Ultra II RNA Library Prep kit. DNA and RNA sequencing was performed by the Scientific Operations core at the WSI on Pacific Biosciences SEQUEL II (HiFi) and Illumina NovaSeq 6000 (RNA-Seq) instruments. Hi-C data were also generated from head and thorax tissue of ilAntChix2 using the Arima v2 kit and sequenced on the Illumina NovaSeq 6000 instrument.

### Genome assembly, curation and evaluation

Assembly was carried out with Hifiasm ( Cheng *et al.*, 2021) and haplotypic duplication was identified and removed with purge_dups ( Guan *et al.*, 2020). The assembly was scaffolded with Hi-C data ( Rao *et al.*, 2014) using YaHS ( Zhou *et al.*, 2023). The assembly was checked for contamination as described previously ( Howe *et al.*, 2021). Manual curation was performed using HiGlass ( Kerpedjiev *et al.*, 2018) and Pretext ( Harry, 2022). The mitochondrial genome was assembled using MitoHiFi ( Uliano-Silva *et al.*, 2022), which performed annotation using MitoFinder ( Allio *et al.*, 2020).

To evaluate the assembly, MerquryFK was used to estimate consensus quality (QV) scores and *k*-mer completeness ( Rhie *et al.*, 2020). The genome was analysed and BUSCO scores ( Manni *et al.*, 2021; Simão *et al.*, 2015) were generated within the BlobToolKit environment ( Challis *et al.*, 2020). Table 3 contains a list of software tool versions and sources.

**Table 3.** Software tools and versions used.

Software tool	Version	Source
BlobToolKit	4.0.7	https://github.com/blobtoolkit/ blobtoolkit
BUSCO	5.3.2	https://gitlab.com/ezlab/busco
Hifiasm	0.16.1-r375	https://github.com/chhylp123/ hifiasm
HiGlass	1.11.6	https://github.com/higlass/higlass
Merqury	MerquryFK	https://github.com/thegenemyers/ MERQURY.FK
MitoHiFi	2	https://github.com/marcelauliano/ MitoHiFi
PretextView	0.2	https://github.com/wtsi-hpag/ PretextView
purge_dups	1.2.3	https://github.com/dfguan/purge_ dups
YaHS	yahs-1.1.91eebc2	https://github.com/c-zhou/yahs

### Ethics and compliance issues

The materials that have contributed to this genome note have been supplied by a Darwin Tree of Life Partner. The submission of materials by a Darwin Tree of Life Partner is subject to the Darwin Tree of Life Project Sampling Code of Practice. By agreeing with and signing up to the Sampling Code of Practice, the Darwin Tree of Life Partner agrees they will meet the legal and ethical requirements and standards set out within this document in respect of all samples acquired for, and supplied to, the Darwin Tree of Life Project. All efforts are undertaken to minimise the suffering of animals used for sequencing. Each transfer of samples is further undertaken according to a Research Collaboration Agreement or Material Transfer Agreement entered into by the Darwin Tree of Life Partner, Genome Research Limited (operating as the Wellcome Sanger Institute), and in some circumstances other Darwin Tree of Life collaborators.

## Data Availability

European Nucleotide Archive: *Antitype chi*. Accession number PRJEB55575; https://identifiers.org/ena.embl/PRJEB55575 ( Wellcome Sanger Institute, 2022) The genome sequence is released openly for reuse. The *Antitype chi* genome sequencing initiative is part of the Darwin Tree of Life (DToL) project. All raw sequence data and the assembly have been deposited in INSDC databases. The genome will be annotated using available RNA-Seq data and presented through the Ensembl pipeline at the European Bioinformatics Institute. Raw data and assembly accession identifiers are reported in Table 1.

## Author information

Members of the Natural History Museum Genome Acquisition Lab are listed here: https://doi.org/10.5281/zenodo.4790042.

Members of the Darwin Tree of Life Barcoding collective are listed here: https://doi.org/10.5281/zenodo.4893703.

Members of the Wellcome Sanger Institute Tree of Life programme are listed here: https://doi.org/10.5281/zenodo.4783585.

Members of Wellcome Sanger Institute Scientific Operations: DNA Pipelines collective are listed here: https://doi.org/10.5281/zenodo.4790455.

Members of the Tree of Life Core Informatics collective are listed here: https://doi.org/10.5281/zenodo.5013541.

Members of the Darwin Tree of Life Consortium are listed here: https://doi.org/10.5281/zenodo.4783558.
