# Supplementary material for: The genome sequence of the Grey Chi, Antitype chi (Linnaeus, 1761)
Source: Wellcome Open Res. 2023 Apr 6;8:157. [Version 1] doi: 10.12688/wellcomeopenres.19288.1 (PMC10477731; doi:10.12688/wellcomeopenres.19288.1)
Supplement: Peer review history

# Peer review: Reviewer response for version 1

**DOI:** 10.21956/wellcomeopenres.21371.r66331

**Published:** 2023-09-04

**Authors:** Jacqueline Heckenhauer

**Affiliations:** 1 Senckenberg Research Institute and Natural History Museum Frankfurt, Frankfurt, Hesse, Germany

In their data note, the authors present ****the genome sequence of the primary assembly and contigs of the alternate haplotype of *Antitype chi *using a *de novo* assembly method following the approach used by the Darwin Tree of Life Project. The presented genome is of high quality and released openly for reuse. Moreover, it is useful to phylogenetic and taxonomic studies (potentially cryptic species). Therefore this high-quality reference genome is very beneficial to the field.

 However, I suggest that the authors include a short summary of repetitive DNA components of the assembly and submit the library of repetitive element (RE) sequences to a public repository of repetitive elements (e.g., Dfam). This way the community would even more benefit from this data. The diversity of available insect genomes has rapidly expanded, but the rate of community contributions to RE databases which are important for RE annotation has not kept pace, preventing high-resolution study of REs in many groups. 

 Was the genome size of this specimen/species estimated by flow cytometry or similar?

Are sufficient details of methods and materials provided to allow replication by others?

Yes

Is the rationale for creating the dataset(s) clearly described?

Yes

Are the datasets clearly presented in a useable and accessible format?

Partly

Are the protocols appropriate and is the work technically sound?

Yes

Reviewer Expertise:

Biodiversity genomics, evolution, trichoptera

I confirm that I have read this submission and believe that I have an appropriate level of expertise to confirm that it is of an acceptable scientific standard.

# Peer review: Reviewer response for version 1

**DOI:** 10.21956/wellcomeopenres.21371.r63289

**Published:** 2023-07-25

**Authors:** Jerome H L Hui

**Affiliations:** 1 The Chinese University of Hong Kong, Hong Kong, China

Lees and colleagues report the genome sequence of a male grey chi *Antitype chi *(Linnaeus, 1761). This work was done as part of the Darwin Tree of Life Project which aims to sequence all named eukaryotic species in the Atlantic Archipelago of Britain and Ireland. This species of moth can be commonly found in Britain. Molecular data of this species are scarce prior to this report, and are mainly confined to COI sequences deposited to the NCBI database. This new genome resource will be very useful for further studies, and to name some, such as identifying potential cryptic species, understanding its roles in the ecosystem, and revealing its evolutionary relationships with other lepidopterans.

 This genome resource is excellent from the summary statistics, with high BUSCO numbers (99.0%), high sequence continuity (scaffold N50), and majority of sequences contained on the 31 pseudochromosomes (plus mitochondrion). To sum up, this is another valuable contribution.

Are sufficient details of methods and materials provided to allow replication by others?

Yes

Is the rationale for creating the dataset(s) clearly described?

Yes

Are the datasets clearly presented in a useable and accessible format?

Yes

Are the protocols appropriate and is the work technically sound?

Yes

Reviewer Expertise:

Genomics, evolution, invertebrates

I confirm that I have read this submission and believe that I have an appropriate level of expertise to confirm that it is of an acceptable scientific standard.
